# The complete mitochondrial DNA sequence of Chuanbai Rex rabbit (*Oryctolagus cuniculus*)

**DOI:** 10.1080/23802359.2020.1848476

**Published:** 2021-01-17

**Authors:** Xiang Wang, Hong Mei Zeng, Yin Wang, Yan Luo, Xue Ping Yao, Zexiao Yang

**Affiliations:** College of Veterinary Medicine, Sichuan Agricultural University, Wenjiang, Sichuan Province, China

**Keywords:** Chuanbai Rex rabbit, *Oryctolagus cuniculus*, mitochondrial genome

## Abstract

Chuanbai Rex Rabbit (*Oryctolagus cuniculus* domesticus) is a hybrid breed in Sichuan, China. In this study, we reveal the mitochondrial genome sequence of the Chuanbai Rex Rabbit for the first time. The length of the mitochondrial genome is 17,179 bp and contains 2 ribosomal RNA genes, 14 protein-coding genes, 22 transfer RNA genes, and 1 D-loop sequence. We further provide a phylogenetic tree showing relationships among Chuanbai Rex Rabbit and other Leporidae species.

The ChuanBai Rex rabbit was bred from Sichuan White Rex rabbit, which originated from German Rex rabbit and American Rex Rabbit and approved by National Livestock and Poultry Genetic Resources Committee (Cheng et al. 2019), moreover ChuanBai Rex rabbit is the first self-bred Rex rabbit new variety in China (Jian et al. 2017). The samples were provided by the Dujiangyan Hongxing Rex rabbit farm (30.9376 N, 103.6627E) and stored in the Animal Quarantine Laboratory of Sichuan Agricultural University.

The mitochondrial genome of ChuanBai Rex rabbitt was extracted by the Mitochondrial Extraction Kit (Beijing Solarbio Science & Technology Co., Ltd). Mitochondrial genome sequencing was performed using prime-walking strategy (Sha et al. 2013) and primer sets for the entire mitochondrial genome amplification and sequencing refer to Sliding Window-Based PSO Algorithm (Yang et al. 2011). The mtDNA sequence was assembled and analyzed by MEGA 7. Protein-coding genes were analyzed by SnapGene v4.1.8.

The complete mtDNA genome of Chuanbai Rex Rabbit has been deposited in GenBank (accession NO. MN953621). Except for ND6, which is located in the light chain, all the other protein-coding genes are located in the heavy chain. For the 22 tRNA, Gln, Ala, Asn, Cys, Tyr, Ser, Glu, Pro were located on the light chain, while the remaining 14 tRNA were located on the heavy chain.The total base composition of the mitochondrial genome is 31.45% A, 28.35% T, 26.54% C and 13.62% G, exhibiting AT bias (59.80%).

The D-loop sequence of the ChuanBai Rex rabbit is very different from the rabbit (*Oryctolagus cuniculus*) reference sequence (accession NO. NC_001913), The D-loop of Chuanbai Rex Rabbit was 1743 bp while the reference sequence was 1800 bp. Repetition of a short 20 bp sequence in the D-loop was repeated for 6 times, while the reference sequence was repeated for 10 times. Repetition of a long 153 bp sequence was unchanged.

A phylogenetic tree was constructed based on the comparison of the complete mitochondrial genome sequences with other Leporidae species by Neighbor joining method (Kumar et al. 2016; Tamura et al. 2004) with Kimura 2-parameter model and for the phylogeny test, bootstrap was set at 1000 (Figure 1).

**Figure 1.**
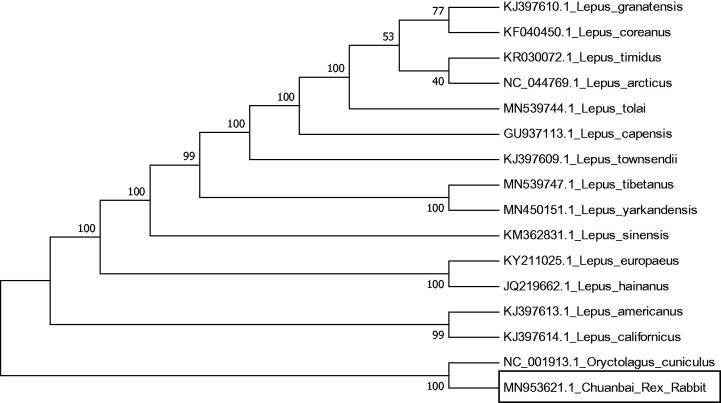
The Neighbor-joining tree based on 14 Lepus and 2 *Oryctolagus cuniculus* complete mitochondrial genome sequences. The numbers at the nodes are bootstrap percent probability values based on 1000 replications.

## Data Availability

The data that support the findings of this study are openly available in NCBI at www.ncbi.nlm.nih.gov, reference number MN953621.
